# Longitudinal determinants of employment status in people with relapsing-remitting multiple sclerosis

**DOI:** 10.1016/j.ibneur.2024.04.002

**Published:** 2024-04-15

**Authors:** E.E.A. van Egmond, K. van der Hiele, M.J. de Rooij, D.A.M. van Gorp, P.J. Jongen, J.J.L. van der Klink, M.F. Reneman, E.A.C. Beenakker, J.J.J. van Eijk, S.T.F.M. Frequin, K. de Gans, E. Hoitsma, O.H.H. Gerlach, J.P. Mostert, W.I.M. Verhagen, L.H. Visser, H.A.M. Middelkoop

**Affiliations:** a Leiden University, Department of Psychology, Health, Medical and Neuropsychology Unit, Leiden, the Netherlands; b Elisabeth-TweeSteden Hospital, Department of Neurology, Tilburg, the Netherlands; c National Multiple Sclerosis Foundation, Rotterdam, the Netherlands; d University of Humanistic Studies, Utrecht, the Netherlands; e Leiden University, Methodology and Statistics Department, Institute of Psychology, Leiden, the Netherlands; f MS4 Research Institute, Ubbergseweg 34, Nijmegen 6522 KJ, the Netherlands; g Department of Community & Occupational Medicine, University of Groningen, University Medical Centre Groningen, PO Box 30001, Groningen 9700 RB, the Netherlands; h Tilburg School of Social and Behavioural Sciences, Tranzo Scientific Centre for Care and Welfare, Tilburg University, PO Box 90153, Tilburg 5000 LE, the Netherlands; i Optentia, North West University of South Africa, PO Box 1174, Vanderbijlspark, South Africa; j Department of Rehabilitation Medicine, Centre for Rehabilitation, University of Groningen, University Medical Centre Groningen, PO Box 30.002, Haren 9750 RA, the Netherlands; k Department of Neurology, Medical Centre Leeuwarden, PO Box 888, Leeuwarden 8901 BR, the Netherlands; l Department of Neurology, Jeroen Bosch Hospital,, PO Box 90153, ‘s-Hertogenbosch 2000 ME, the Netherlands; m Department of Neurology, St. Antonius Hospital, PO Box 2500, Nieuwegein 3430 EM, the Netherlands; n Department of Neurology, Groene Hart Hospital, PO Box 1098, Gouda 2800 BB, the Netherlands; o Department of Neurology, Alrijne Hospital, PO Box 4220, Leiderdorp, the Netherlands; p Department of Neurology, Zuyderland Medical Centre, PO Box 5500, Sittard-Geleen 6130 MB, the Netherlands; q Department of Neurology, Maastricht University Medical Centre, PO Box 5800, Maastricht 6202 AZ, the Netherlands; r Department of Neurology, School for Mental Health and Neuroscience, Maastricht University Medical Centre, PO Box 5800, Maastricht 6202 AZ, the Netherlands; s Department of Neurology, Rijnstate Hospital, PO Box 9555, Arnhem 6800 TA, the Netherlands; t Department of Neurology, Canisius-Wilhelmina Hospital, PO Box 9015, Nijmegen 6500 GS, the Netherlands; u Leiden University Medical Centre, Department of Neurology & Neuropsychology, Leiden, the Netherlands

**Keywords:** Multiple sclerosis, Employment, Work, Depression, Fatigue, Cognition

## Abstract

**Purpose:**

To investigate longitudinal relationships between employment status and disease-related, (neuro)psychological, and work-related factors in people with multiple sclerosis (MS).

**Methods:**

170 employed people with MS underwent yearly neurological and neuropsychological examinations to assess MS-related disability and cognitive functioning. Additionally, they completed yearly questionnaires assessing depression, anxiety, fatigue, cognitive complaints, workplace support and coping. Multilevel models for change were fitted to examine progression of these factors over three years, and to assess possible relationships with change in employment status.

**Results:**

People with a deteriorated employment status after three years reported more depression (*p=*0.009), a higher impact of fatigue (*p<*0.001), more cognitive complaints (*p<*0.001) and less workplace support (*p=*0.001) at baseline than people with a stable employment status. There were no differences in progression over time of the examined variables between people with a stable or deteriorated employment status.

**Conclusion:**

More depression, a higher impact of fatigue, more cognitive complaints and less workplace support are predictive of a deteriorated employment status after three years in individuals with MS. How these factors progress over time is not different between those with a stable or deteriorated employment. MS-related disability, anxiety, objective cognition and coping were not related to a deterioration in employment status.

## Highlights

• Symptoms of depression are related to a change in work status in MS.
• Cognitive complaints and fatigue predict deterioration in work status in three years.
• Less workplace support predicts a deteriorated work status in three years.

## 1 Introduction

There is strong evidence to suggest that multiple sclerosis (MS) negatively impacts employment status (Schiavolin et al., 2013). Research evaluating the role of MS in employment has gone through major changes. While earlier MS research regarded measures of work participation a secondary outcome (Schiavolin et al., 2013), current research justly considers work measures a primary outcome. Research on job retention is paramount given the financial consequences (Kobelt et al., 2017) and the effect of job loss on mental wellbeing in people with MS. Research indicates that early retiring due to disability in MS is negatively associated with mental health related quality of life (Marck et al., 2020).

The loss of employment in this population is the consequence of reciprocal relationships between disease-related factors, personal factors and contextual factors (Meide et al., 2018). Disease-related factors such as physical disability and fatigue have often been linked to employment status (Boe Lunde et al., 2014, Conradsson et al., 2020, D'Hooghe M et al., 2019, Kobelt et al., 2019, Krause et al., 2013, Oliva Ramirez et al., 2021, Raggi et al., 2016, Salter et al., 2017, Schiavolin et al., 2013, Simmons et al., 2010). However, these factors only offer a partial explanation for unemployment rates in people with MS (Dorstyn et al., 2019).

Furthermore, personal factors such as (neuro)psychological characteristics are crucial in the stability of employment (Dorstyn et al., 2019), and multiple, predominantly cross-sectional studies have been carried out on the effect of depression, anxiety, coping styles and cognition. Several studies showed an association between more symptoms of depression and anxiety and unemployment (Conradsson et al., 2020, Dorstyn et al., 2019, Krokavcova et al., 2010, Povolo et al., 2019, Raggi et al., 2016), but not all (D'Hooghe M et al., 2019, Hartoonian et al., 2015, Povolo et al., 2019, Smith and Arnett, 2005). Moreover, coping styles have been linked to employment. While an avoidant related coping style is often associated with worse work outcomes such as unemployment, a problem focused coping style has a positive impact on employment status (Dorstyn et al., 2019, Grytten et al., 2017, Holland et al., 2019, Strober and Arnett, 2016, Vijayasingham and Mairami, 2018). With respect to cognition, both subjective and objective measures have been linked to employment status (D'Hooghe M et al., 2019, Morrow et al., 2010, van Gorp et al., 2019).

In addition, contextual factors such as the work environment might contribute to employment outcomes in MS (Vijayasingham and Mairami, 2018, Vitturi et al., 2022). A recent meta-analysis highlighted the need of further research analysing the impact of a supportive and inclusive work environment specifically (Dorstyn et al., 2019). Previous qualitative research (Meide et al., 2018) identified facilitators and barriers of employment through interviews with people with MS. One of the core themes was “an understanding line manager”. A manager that takes care of the well-being of the employee, and is capable of assessing both limitations and capabilities of their employees with MS, is essential in job retention. Additionally, research (Honan et al., 2012) showed that experiencing a non-supportive work environment increased the proportion of work hours reduced since the diagnosis as well as the likelihood of withdrawing from work and changing type of work.

However, the majority of the aforementioned studies are cross-sectional in nature. A recent meta-analysis identified the need for longitudinal data to identify characteristics of people maintaining employment to clarify possible causal pathways (Gerhard et al., 2020). Therefore, the current study aims to assess relationships between disease-related, (neuro)psychological and work contextual factors, and employment status using a longitudinal design, while controlling for demographic factors. Specifically, we aim to 1) identify people at risk for a deterioration in employment status, by examining differences at baseline between people who have a stable employment status and those who do not and 2) examine whether the progression of relevant variables differs over time between the people who have a stable employment status and those who do not. In order to investigate this we adopt a longitudinal growth trajectory approach. Only few studies have adopted this longitudinal growth trajectory approach to examine disease-related factors in MS (Veldhuijzen van Zanten et al., 2021), enabling the analysis of within-person variance. Such an approach enables inclusion of multiple data points (>2) and enables us to ascertain whether the variables of interest fluctuate differently over time between the stable employment group and the deteriorated employment group.

With respect to disease-related factors, we hypothesise that less MS-related disability, better objective cognitive functioning and a lower impact of fatigue at baseline will be related to a stable employment status within a period of three years. Additionally, we hypothesise that less symptoms of depression and anxiety, less cognitive complaints, less frequent use of emotion-related and avoidance-related coping, and a more frequent use of task-related coping at baseline will be related to a stable employment status over three years. To assess the influence of the work context, we examined the extent to which people with MS experienced a supportive workplace. We hypothesise that a less supportive workplace will be associated with a deterioration in employment status within three years. Finally, concerning the progression over time (growth trajectories), we expect a larger decrease in objective cognitive functioning, task-oriented coping and workplace support to be related to a deterioration in employment status. We expect an increase over time in MS-related disability, fatigue, depression and anxiety, cognitive complaints, avoidance and emotion oriented coping to be related to a deteriorated employment status.

## 2 Materials and methods

### 2.1 Participants

**Fig. 1:**
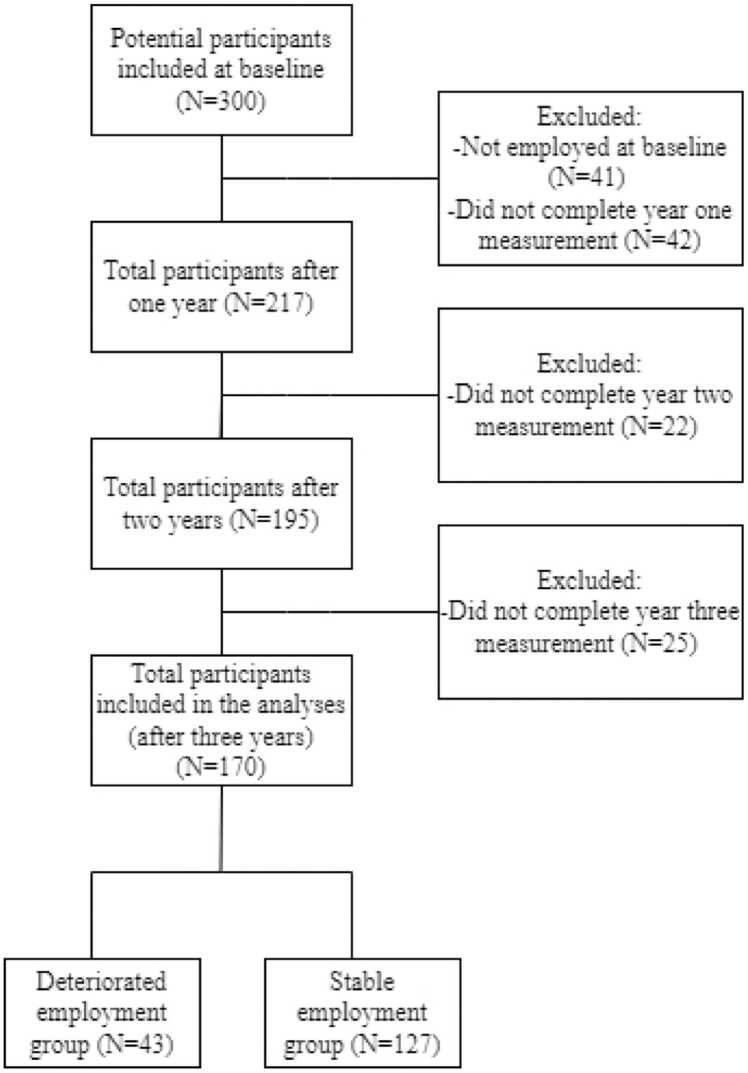
Flowchart participants.

300 potential participants were recruited for the MS@Work study, a three-year prospective observational study aimed to identify predictors of work participation in people with relapsing-remitting MS (van der Hiele et al., 2015). The inclusion requirements were being 18 years or older, having a relapsing-remitting MS diagnosis according to the Polman-McDonald criteria (Polman et al., 2011), being proficient in the Dutch language, and being in paid employment or within three years since the last employment. People who were diagnosed with comorbid neurological or neuropsychiatric disorders or substance abuse were not approached to participate. For the current study we selected only people who were employed at baseline (259 people), to establish the change in employment status. Ultimately, 170 people who were employed at baseline finished measurements after three years resulting in a study sample of 170 people (See Fig. 1. for a flowchart of the inclusion of participants).

The current study was approved by the Medical Ethical Committee Brabant (NL43098.008.12 1307), and all participants signed an informed consent form before participation.

### 2.2 Procedure

At baseline, and after one, two, and three years participants were asked to complete online questionnaires concerning the impact of fatigue, depression, anxiety, cognitive complaints, coping styles, work-related variables and demographic characteristics. Additionally, participants underwent yearly neurological and neuropsychological assessments to examine objective cognitive functioning and MS-related disability. All measurements were included in the statistical analyses.

### 2.3 Materials

#### 2.3.1 Disease related factors

We used the Modified Fatigue Impact Scale (MFIS) to examine the impact of fatigue (Kos et al., 2003). The total score ranges from 0 to 84, with a higher score being indicative of a higher impact of fatigue.

To assess MS-related disability, the Expanded Disability Status Scale (EDSS) score was assessed by an experienced neurologist (Kurtzke, 1983). This scale ranges from 0 to 10, with higher scores reflecting a higher disability.

#### 2.3.2 (Neuro)psychological factors

In order to examine symptoms of depression and anxiety, we used the Hospital Anxiety and Depression Scale (HADS) (Spinhoven et al., 1997). This scale consists of 14 items, 7 on depression and anxiety respectively. Scores on both subscales range from 0 to 21. A higher score means more symptoms of depression or anxiety.

We used the Coping inventory for Stressful Situations (CISS) (De Ridder and v. H., 2004, Endler and Parker, 1999) to assess preferred coping styles. The CISS distinguishes three main coping styles: task-oriented coping, emotion-oriented coping and avoidance-oriented coping. The scores on each subscale range from 16 to 80. A higher score indicates a more frequent usage of that particular coping style.

The MS Neuropsychological Screening Questionnaire (MSNQ) (Benedict et al., 2003) was used to screen for cognitive complaints. The total score ranges from 0 to 60, with a higher score being reflective of more cognitive complaints.

The Symbol Digit Modalities Test (SDMT) (Smith, 1982) (written version) was chosen as a measure of objective cognitive functioning, visual information processing speed in particular. Possible total scores range from 0 to 110, with higher scores being reflective of a higher information processing speed (i.e. better objective cognitive functioning).

#### 2.3.3 Work context

To measure the extent to which the workplace was considered supportive, we used the ‘non-supportive workplace’ subscale of the Multiple Sclerosis Work Difficulties Questionnaire (MSWDQ) (Honan et al., 2012). Possible scores range from 0 to 100, with a higher score indicating less experienced support in the workplace.

In order to examine employment status we asked participants yearly whether they were employed. Based on the subsequent measurements we assessed whether the employment status remained stable or had deteriorated over three years. We considered employment status as deteriorated (deteriorated employment status; DES) if someone quit their job or decreased their work hours due to MS (Morrow et al., 2010). People who did not report any changes with respect to being employed or the amount of work hours were characterized as having a stable employment status (SES). People who increased their work hours were included in the stable employment group. Employment status (SES/DES) was added as a predictor to retrospectively identify people at risk for a deterioration in employment status.

#### 2.3.4 Demographic characteristics

We asked participants for their age, gender and educational level. Educational level was divided into three categories: lower education (completed low-level secondary school), middle education (completed secondary school medium level) and higher education (completed secondary school at the highest level).

### 2.4 Statistical analyses

Multilevel models for change (Singer, 2003) were fitted to examine progression over time, and to assess possible relationships with change in employment status. Models were created for anxiety, depression, impact of fatigue, MS-related disability, cognitive complaints, objective cognitive functioning, coping styles, and workplace support. The used analysis approach constitutes of fitting four models for each of the above mentioned variables. Firstly, we fitted an unconditional means model (UMM) to assess the intraclass correlation coefficient (the degree of variability between groups). Second, an unconditional growth model (UGM) was fitted to verify whether there are individual differences in starting point (the score at baseline) and progression over time. Thirdly, in the conditional growth model (CGM) we added demographic and disease related factors, i.e. gender, age, education, disease duration, and its interaction with time. Finally, we added change in employment status and its interaction with time as fixed effects to examine whether the SES/DES group differ at baseline and/or over time. Likelihood ratio tests were used for model comparison. To increase readability only the fourth model will be included in the manuscript. The first through the third model will be included in the supplementary material. Tables are included in the manuscript for models in which work is a significant correlate, while other tables were included in the supplementary material.

Multilevel models were fitted using R (R Core Team, R Foundation for Statistical Computing, Vienna, Austria), using the lme4 (Bates, 2015) and lmerTest packages (Satterthwaite’s method) (Kuznetsova, 2017). Plots were made using the ggplot package (Wickham, 2009). Values of *p*≤0.05 were considered as significant.

## 3 Results

### 3.1 Participants

For the current study we selected only people who were employed at baseline (259 people), and finished the three year measurements resulting in a sample of 170 people with relapsing-remitting MS. There were 127 people in the SES group, of which 118 people were in paid employment and 9 people were self-employed. The deteriorated employment status (DES) group comprised 43 people, of which 24 people reported working less hours, and 19 people reported job loss after three years. Sample characteristics at baseline are listed in Table 1, and the progression over time is presented in Table 2.

**Table 1:** Sample characteristics at baseline.

	Total sample *N*=170	SES *N*=127	DES *N*=43
Gender (%female)^a^	138 (81.2%)	100 (78.7%)	38 (88.4%)
Age^b^	42.00 (9.30)	41.59 (9.01)	43.21 (10.11)
Educational level (N, %)			
Lower^a^	24 (14.1%)	15 (11.8%)	9 (20.9%)
Middle^a^	64 (37.6%)	52 (40.9%)	12 (27.9%)
Higher^a^	82 (48.2%)	60 (47.2%)	22 (51.2%)
Work hours per week^c^	28 (18)	28 (20)	24 (20)
Disease duration (y)^c^	5.8 (8.08)	5.5 (7.42)	7.2 (11.28)
MS-related disability (EDSS)^c^	2.0 (1.0)	2.0 (1.0)	2.5 (1.5)
Anxiety^c^ (HADS)	5.0 (4.00)	5.0 (4.00)	5.0 (3.00)
Depression^c^(HADS)	2.0 (3.00)	2.0 (2.00)	3.5 (5.00)
Fatigue^b^ (MFIS)	33.6 (14.96)	31.3 (14.63)	40.4 (13.96)
Cognitive complaints (MSNQ)^c^	22.0 (15.00)	20.0 (15.00)	28.5 (12.50)
Objective cognition (SDMT)^c^	55.5 (9.00)	56 (9.75)	53.5 (15.25)
Supportive workplace^c^ (MSWDQ)	5.0 (15.00)	5.0 (15.00)	10.0 (30.00)
Task-oriented coping^c^ (CISS)	61.0 (9.00)	61.0 (8.75)	60.0 (9.50)
Emotion-oriented coping^b^ (CISS)	36.1 (10.25)	36.4 (10.33)	35.4 (10.08)
Avoidance-oriented coping^b^ (CISS)	46.3 (9.00)	45.9 (9.18)	47.4 (8.42)

SES=Stable employment status, DES= Deteriorated employment status. EDSS= Expanded Disability Status Scale. HADS= Hospital Anxiety and Depression Scale. MFIS= Modified Fatigue Impact Scale MSNQ= Multiple Sclerosis Neuropsychological Screening Questionnaire. SDMT= Symbol Digit Modalities Test. MSWDQ=MS Work difficulties Questionnaire. CISS= Coping inventory for Stressful Situations.

a N(%).

b Mean (Standard Deviation).

c Median (Inter Quartile Range); in case of not normally distributed data.

**Table 2:** Three-year changes in disease-related, (neuro)psychological and work contextual factors for people with a stable and deteriorated employment status.

	Baseline		Year 1		Year 2		Year 3	
	SES (N=127)	DES (N=43)	SES	DES	SES	DES	SES	DES
MS-Related disability EDSS^b^	2.0 (1.0)	2.5 (1.5)	2.00 (1.5)	2.5 (1.3)	2.0 (1.5)	2.5 (1.3)	2.0 (1.5)	2.0 (1.0)
HADS Anxiety^b^	5.0 (4.00)	5.0 (3.00)	4.0 (4.00)	5.0 (3.05)	5.0 (4.00)	4.5 (3.25)	4.0 (3.00)	4.5 (2.25)
HADS Depression^b^	2.0 (2.00)	3.5 (5.00)	2.0 (3.00)	3.5 (4.00)	2.0 (3.00)	4.0 (5.00)	2.0 (3.00)	3.5 (4.50)
Fatigue^a^	31.3 (14.63)	40.4 (13.96)	29.9 (15.04)	44.3 (11.98)	29.4 (14.70)	43.5 (12.82)	29.4 (14.67)	41.8 (12.43)
Cognitive complaints^b^	20.0 (15.00)	28.5 (12.50)	18.0 (12.75)	30.0 (17.25)	20.0 (9.75)	29.0 (14.00)	21.0 (12.75)	31.5 (12.50)
Objective cognition^b^	56.0 (9.75)	53.5 (15.25)	56.0 (8.33)	52.0 (16.25)	56.0 (13.25)	52.5 (15.25)	57.0 (14.00)	54.0 (14.50)
Supportive workplace^b^	5.0 (15.00)	10.0 (30.00)	0.0 (10.00)	8.0 (20.00)	0.0 (15.00)	10.0 (25.00)	0.0 (10.00)	2.0 (13.00)
Task-oriented coping^b^	61.0 (8.75)	60.0 (9.50)	60.0 (9.75)	57.0 (10.50)	60.0 (10.00)	57.0 (7.25)	60.0 (11.00)	55.0 (10.25)
Emotion-oriented coping^a^	36.4 (10.33)	35.4 (10.08)	33.1 (9.73)	34.7 (10.59)	33.6 (10.71)	31.4 (10.00)	32.2 (10.47)	33.4 (10.43)
Avoidance-oriented coping^a^	45.9 (9.18)	47.4 (8.42)	45.3 (9.98)	45.9 (9.40)	44.4 (8.91)	45.3 (9.35)	45.3 (10.01)	45.5 (10.69)

SES=Stable employment status. DES=Deteriorated employment status ^a^Mean (Standard deviation).^b^Median (Interquartile range); in case of not normally distributed data.

### 3.2 Multilevel models for EDSS

#### 3.2.1 CGM (Model 4)

In model 4, we added change in employment status and its interaction with time to the model (See supplemental Table 1). Model 4 significantly improved the fit of the model compared to Model 3 (χ^2^(2)=6.62, *p=*0.037), and thus was considered the best model. This model explained 16.7% of the variance of the intercepts of the UGM and 1.7% of the slopes when compared to the UGM. Age at baseline and disease duration were positively significantly related to MS-related disability. The DES group had a higher EDSS at baseline (estimated difference is 0.31), and the score increased more rapidly with time (estimated difference is 0.10 per year). However, these effects were not statistically significant. Whereas the increase in EDSS is 0.02 per year for the SES group, the increase is 0.02 + 0.10 per year for the DES group. The direct effect of time was not significant.

### 3.3 Multilevel models for anxiety

#### 3.3.1 CGM (Model 4)

Fitting a CGM (Model 4; adding change in employment status SES/DES, and its interaction with time) did not improve the model fit compared to model 3 (*χ*^*2*^(2)=2.13, *p=*0.346). Model 4 explained no more variance in the intercepts compared to the UGM, but did explain 7% more variance in the slopes. The interaction between time and gender was positively significant in model 4 (*p=*0.006). Employment status was not significantly associated with anxiety. Neither time nor the interaction between time and employment status was significantly associated with anxiety. The fixed effect estimates and associated statistics of this model are given in Supplemental Table 2.

### 3.4 Multilevel models for depression

#### 3.4.1 CGM (Model 4)

Fitting a CGM (Model 4; adding change in employment status SES/DES and its interaction with time) did not improve the model fit compared to model 3 (*χ*^*2*^(2)=2.13, *p=*0.3455). Employment status is, however, significantly associated with depression. The DES group had a higher depression score at baseline (estimated difference is 1.18; See Table 3). Time was significantly associated to depression, indicating that depression scores increased over time (0.93, t = 2.06, p = 0.041). Additionally, the interaction between time and age on depression was significant, that is the time effect goes down with age (-0.02, t = −2.03, p = 0.044). The interaction effect of time and employment status was not significantly related to depression. Adding employment status resulted in 5% explained variance in the slopes of fatigue (compared to the unconditional growth model), but did not explain additional variance for the intercepts (0%).

**Table 3:** Fixed effects for the CGM (Model 4) of depression.

	Estimate	Standard error	t-value	p-value
Intercept	2.55	1.31	1.95	0.053
Time	0.93	0.45	2.06	**0.041**
DES	1.18	0.45	2.61	**0.009**
Age at baseline	0.02	0.02	0.72	0.473
Disease duration baseline	-0.06	0.03	-1.62	0.108
Education	-0.13	0.28	-0.44	0.660
Gender (male)	0.31	0.50	0.62	0.536
Time*DES	0.04	0.15	0.24	0.812
Time* Age	-0.02	0.01	-2.03	**0.044**
Time*Disease duration	0.00	0.01	0.21	0.835
Time*Education	-0.07	0.10	-0.78	0.438
Time*Gender (male)	0.30	0.17	1.76	0.080

Bold values indicate significant p-values.

### 3.5 Multilevel models for the impact of fatigue

#### 3.5.1 CGM (Model 4)

Fitting a CGM (Model 4; adding change in employment status SES/DES and its interaction with time) did improve the model fit compared to model 3 (*χ*^*2*^(2)=25.09, *p=*3.558*10^−06^). Adding employment status resulted in 12% explained variance in the intercepts of fatigue (compared to the unconditional growth model), but no explained variance for the slopes (0%).^1^ Both employment status and gender were significantly associated with the impact of fatigue (see Table 4). The DES group reported a higher impact of fatigue score at baseline (estimated difference is 10.19). Women reported a higher impact of fatigue than men. Neither time nor the interaction of time and employment status was significantly related to the impact of fatigue.

**Table 4:** Fixed effects for the CGM (Model 4) of the impact of fatigue.

	Estimate	Standard error	t-value	p-value
Intercept	31.84	7.21	4.42	1.820*10^**−05**^
Time	0.72	1.94	0.37	0.713
DES	10.19	2.49	4.09	6.66*10^**−05**^
Age at baseline	0.08	0.14	0.62	0.538
Disease duration baseline	-0.20	0.19	-1.03	0.304
Education	-0.67	1.57	-0.43	0.669
Gender (male)	-6.04	2.76	-2.19	**0.030**
Time*DES	0.84	0.66	1.28	0.201
Time* Age	-0.02	0.04	-0.43	0.668
Time*Disease duration	-0.00	0.05	-0.13	0.900
Time*Education	-0.24	0.41	-0.59	0.555
Time*Gender (male)	0.575	0.723	0.800	0.427

Bold values indicate significant p-values.

### 3.6 Multilevel models for cognitive complaints

#### 3.6.1 CGM (Model 4)

Fitting a CGM (Model 4; adding change in employment status SES/DES) did improve the model fit compared to model 3 (*χ*^*2*^(2)=26.68, *p=*1.608*10^−06^). Including employment status lead to 12% explained variance of the intercepts, but no explained variance of the slopes. Employment status, educational level and the interaction between time and age were significantly associated with cognitive complaints (see Table 5). The DES group scored higher on cognitive complaints than the SES group at baseline (estimated difference is 6.86). Neither time nor the interaction effect of time and employment status was significantly related to cognitive complaints.

**Table 5:** Fixed effects for the CGM (Model 4) of cognitive complaints.

	Estimate	Standard error	t-value	p-value
Intercept	23.28	4.89	4.76	4.17*10^**−06**^
Time	2.50	1.41	1.77	0.079
DES	6.86	1.69	4.07	7.43*10^**−05**^
Age at baseline	0.11	0.09	1.16	0.249
Disease duration baseline	-0.24	0.13	-1.85	0.066
Education	-2.14	1.06	-2.02	**0.045**
Gender (male)	0.43	1.87	0.23	0.819
Time*DES	0.77	0.48	1.63	0.106
Time* Age	-0.06	0.03	-2.07	**0.041**
Time*Disease duration	0.02	0.04	0.67	0.508
Time*Education	-0.19	0.30	-0.64	0.527
Time*Gender (male)	0.57	0.53	1.09	0.279

Bold values indicate significant p-values.

### 3.7 Multilevel models for objective cognition

#### 3.7.1 CGM (Model 4)

Fitting a CGM (Model 4; adding change in employment status SES/DES) did not improve the model fit compared to model 3 (*χ*^*2*^(2)=4.06, *p=*0.131). Including employment status lead to 15% explained variance of the intercepts, and 7% explained variance of the slopes compared to Model 2. Time, age and the interaction between time and age were significantly associated with objective cognition (see Supplementary Table 3). A younger age was associated with a better objective cognition score. Time was positively related to cognition, indicating higher scores on the SDMT over time (2.07, t = 2.03, p = 0.044). Employment status and the interaction between time and employment status were not significantly associated with objective cognition. Using standardized norm scores (controlling for age and educational level and converted to z-scores) did not yield different outcomes.

### 3.8 Multilevel models for supportive workplace

#### 3.8.1 CGM (Model 4)

Fitting a CGM (Model 4; adding change in employment status SES/DES) did improve the model fit compared to model 3 (*χ*^*2*^(2)=11.47, *p=*0.003). Including employment status, the percentage of explained variance of the intercepts is 11%, and 2% of the slopes (compared to the UGM). Employment status was significantly associated with a supportive work environment (see Table 6). The DES group had a higher score on the workplace support scale (indicating less perceived support) at baseline (estimated difference is 6.33). Neither time nor the interaction between time and employment status was significantly related to workplace support.

**Table 6:** Fixed effects for the CGM (Model 4) of workplace support.

	Estimate	Standard error	t-value	p-value
Intercept	20.99	6.87	3.06	**0.003**
Time	-1.85	2.90	-0.64	0.526
DES	7.91	2.36	3.34	**0.001**
Age at baseline	-0.14	0.13	-1.05	0.295
Disease duration baseline	-0.06	0.18	-0.34	0.732
Education	-2.59	1.49	-1.74	0.083
Gender (male)	-1.18	2.62	-0.45	0.653
Time*DES	-1.38	0.98	-1.40	0.163
Time* Age	0.00	0.05	-0.02	0.983
Time*Disease duration	0.10	0.07	1.39	0.168
Time*Education	0.32	0.62	0.51	0.610
Time*Gender (male)	1.31	1.08	1.21	0.227

Bold values indicate significant p-values.

### 3.9 Multilevel models for task-oriented coping

#### 3.9.1 CGM (Model 4)

In model 4, we added change in employment status and its interaction with time to the model. Model 4 did not significantly improve the fit of the model as compared to model 3 (χ^2^(2)=2.76, *p=*0.252). Educational level is positively significantly associated with task-oriented coping (see Supplemental Table 4). Time and the interaction between time and employment status were not significantly related to task-oriented coping.

### 3.10 Multilevel models for emotion-oriented coping

#### 3.10.1 CGM (Model 4)

In model 4, we added change in employment status and its interaction with time to the model (See Supplemental Table 5). Model 4 did not significantly improve the fit of the model as compared to model 3 (χ^2^(2)=0.55, *p=*0.761). Model 4 did not explain more variance in either the slopes or the intercepts compared to the UGM (0%). Neither time nor the interaction between time and employment status was significantly associated with emotion-oriented coping.

### 3.11 Multilevel models for avoidance oriented coping

#### 3.11.1 CGM (Model 4)

In model 4, we added change in employment status and its interaction with time to the model. Model 4 did not significantly improve the fit of the model (χ^2^(2)=1.26, *p=*0.532). The interaction between time and disease duration (*p=*0.043) and the interaction between time and gender (*p=*0.002) were significantly associated with avoidance-oriented coping (positively and negatively respectively; See Supplemental Table 6). Neither time nor the interaction between time and employment status was significantly related to avoidance-oriented coping.

## 4 Discussion

The current study aimed to examine the longitudinal relationship between employment status and disease-related, (neuro)psychological and work-related factors in people with MS. We first examined baseline differences between people with MS with a stable or deteriorated employment status. Additionally, we explored the growth trajectories per factor and analysed whether these trajectories differed between people with a stable or deteriorated employment status. We demonstrated that more symptoms of depression, a higher impact of fatigue, more cognitive complaints and less workplace support at baseline were related to a deterioration of employment status within three years. MS-related disability, anxiety, objective cognition and coping styles were not related to a deterioration in employment status. Moreover, the progression over time (growth trajectories) of any of the disease-related, neuropsychological or work-related factors did not differ between people with a stable or deteriorated employment status.

### 4.1 Employment status in relation to (neuro)psychological factors

In line with earlier research (Dorstyn et al., 2019, Raggi et al., 2016), more depressive symptoms were associated with a deteriorated employment status within three years, even though the median scores were noticeably low in the current sample. Only 7% of the participants had scores that were indicative of depression (which is similar to the 8.5% prevalence in the general Dutch population (Ten Have et al., 2023). The precise mechanisms underlying this relationship have not yet been identified. Previous research has often linked depressive symptoms to negative biases (LeMoult, 2019), which may affect the perception of the work situation. Another possible explanation might be the role of hope. Research by Lynch and colleagues (Lynch et al., 2001) suggests that there might be a negative relationship between experiencing hope and depression. Recently, preliminary evidence was found suggesting that people with MS who experience more hope have higher odds of being in employment (Lee et al., 2022). Factors such as depression and hope might be modifiable, thereby being relevant aspects to integrate in interventions.

As was found in previous research a higher impact of fatigue was related to a deterioration in employment status. Fatigue is one of the most common reported complaints in MS, and there is substantial evidence that experiencing fatigue is related to worse work participation outcomes (Oliva Ramirez et al., 2021).

Subjective cognitive problems (i.e. cognitive complaints) were associated with having a deteriorated employment status, while objective cognitive problems were not. Both objective and subjective measures of cognition have been linked to employment measures in previous research, despite the weak correlation between subjective and objective cognitive functioning (Morrow et al., 2010, van Gorp et al., 2019). Interestingly, previous research suggests that subjective cognitive difficulties may be more related to depression, fatigue, anxiety and self-efficacy than objective cognitive measures. Depression is hypothesised to alter the perception of cognitive difficulties (Strober et al., 2016). Possibly, this trend may also be present for work participation and these factors may contribute to decision making with regard to work maintenance. In addition, the current sample was characterised by relatively spared objective cognition, which may explain why work decisions are more related to subjective changes in the current sample.

### 4.2 Employment status in relation to work-related factors

The previously mentioned factors are all personal factors. However, the current results suggest that the workplace also has a part to play in job retention. Specifically, experiencing less support from the workplace was related to a deterioration in employment status. Qualitative research has argued that environmental factors might be equally relevant as disease-related factors when considering employment (De Dios Pérez, 2022). When asked, working people with MS identified co-workers’ attitudes as one of the most difficult aspects of the workplace. In particular, a lack of understanding in colleagues and line managers on the subject of MS was perceived as an issue. This finding relates to a recent systematic review by Vitturi and colleagues (Vitturi et al., 2022) in which they suggest that stigma and discrimination can discourage people with disabilities from pursuing employment or maintaining it. Additionally, perceived stigma and/or discrimination may prevent employees from disclosure of their MS diagnosis which in turn may hinder the usage of appropriate accommodations in the work setting.

### 4.3 Employment status in relation to disease-related factors

Interestingly, in the current study we did not find evidence for a difference in MS related disability (EDSS) between the DES and SES group, as opposed to previous research. This may be due to the current sample that is characterised by having relatively limited disability.

### 4.4 Employment status in relation to growth trajectories of disease-related, (neuro)psychological and work-related factors

In contrast to our hypotheses, we did not see differences in the progression over time (growth trajectories) of the individual factors between the stable and deteriorated group. In the current sample the majority of the examined variables remained relatively stable over a period of three years. These findings may reflect a stable sample, but arguably the current design is not suitable to detect such changes, e.g. due to too infrequent measurements or a too short time period (three years).

### 4.5 Implications for future research

While current research illuminates several relevant individual factors, it needs to be acknowledged that the amount of explained variance per variable is low. This is not surprising, given that work participation needs to be considered as a multifactorial issue. This notion is in line with the Work Disability Prevention Model (Loisel et al., 2001). This model was initially developed to analyse factors contributing to the process of returning to work for people with low back pain. Recently the model has proven to be insightful in the concept of staying at work as well, and has been applied in several patient populations (Dijkstra et al., 2023). The model adopts a holistic approach and acknowledges four separate systems that contribute to work, being the workplace, personal factors, healthcare factors and legislative/insurance related factors, as well as overarching factors. Relevant factors can be mapped within these categories for individual workers (Dijkstra et al., 2023). Future research should aim to map relevant factors within these categories to identify possible important factors that have not yet been addressed in the field of MS.

Moreover, it would be insightful to replicate the current study in people who were recently diagnosed with MS. Although the current study takes disease duration into account, we know from previous research that the time period directly after receiving the diagnosis is important for making relevant life choices. For instance, it has been reported that 43% of the people with MS who leave the workforce do so within three years after receiving their diagnosis (Jones, 2016). Given that the application of multilevel modelling benefits from a ‘substantively meaningful metric for time’ (Singer, 2003), it might be beneficial to adopt a multilevel approach in this group of people to be able to intervene timely and prevent job termination.

### 4.6 Implications for clinical practice

Momsen et al. created an overview of reviews on rehabilitation in MS and concluded that vocational rehabilitation should be initiated early to identify barriers and tackle the effect of MS-related symptoms (Momsen et al., 2022). The current study found effects of fatigue, depression, cognitive complaints and workplace support on employment status. These are all ‘invisible symptoms’ which may be hard to grasp in clinical practice. Additionally, symptoms in the current sample may appear subtle. For instance, while the average scores for cognitive complaints and fatigue in the DES group are above the clinical cut-off scores, the average score for depression is not clinically significant. Therefore, rather than overmedicalizing people, individually tailored, guided exercise training can be considered to decrease feelings of fatigue and depression (Momsen et al., 2022). In addition, there is positive evidence that suggests that physical exercise can also be used to decrease cognitive difficulties (Sandroff et al., 2016), however more well-designed studies need to be carried out to definitely confirm this relationship. In addition, it is important for health care professionals to ascertain the amount of support that an individual with MS is currently receiving, both within and outside the work context. When needed, patients should be able to utilise resources such as vocational rehabilitation, job coaches, guidance from an occupational health physician or mental health professional to increase feelings of support and promote self-efficacy.

However, as mentioned above, MS-related factors cannot be considered in isolation, and one size does not fit all. An individual with his/her own values, preferences and difficulties needs to be considered within a specific work setting within a bigger context. These analyses require an interdisciplinary patient-centered approach, including an occupational health physician, to tackle MS-related symptoms, and share knowledge on possible accommodations and legislations to facilitate a coordinated treatment and return to work/ stay at work plan.

### 4.7 Strengths and limitations

A strength of the current research is the longitudinal design, enabling the examination of clinical variables over the course of time. To the best of our knowledge, the current study is one of few studies applying a multilevel approach to clinical data of people with MS. This method enables integration of within-person variation to examine possible fluctuations over time, obtaining robust and clinically relevant information (Veldhuijzen van Zanten et al., 2021).

On the other hand, several limitations should be recognized. Firstly, the current sample consists of people with MS with relatively limited disability (Median EDSS=2.0), and the employment rate was high when compared to other studies incorporating work measures and mental health (Dorstyn et al., 2019). Moreover, participants scored low on measures such as depression and anxiety (below the clinical cut-off) and workplace support. These sample characteristics may be the result of a selection effect, and it could be challenged whether the current sample is representative of the entire MS population, raising the question of generalization. On the other hand, the current sample might be reflective of the specific clinical population working in the Netherlands. When compared to neighbouring countries, the Netherlands has a lower employment rate for people with MS, probably due to a relatively generous invalidity benefit. Moreover, people with MS more frequently work part time (Uitdehaag et al., 2017). These societal factors may impact decisions regarding work. The current results in this sample, characterised by limited disability, might therefore be particularly relevant for preventive occupational care. Future research should also include the type of work people do, given that job type might influence the feasibility of job maintenance.

Secondly, the current study excluded people who were not proficient in Dutch, excluding people with low literacy. This tendency is often seen in clinical research and needs to be tackled to improve generalization to the entire population.

Thirdly, we included 259 employed people with MS at baseline. 170 people completed the three-year-measurements, indicating a dropout rate of 34% (as opposed to the expected 10% (van der Hiele et al., 2015) possibly affecting the validity of the results. Participants were compared to the drop out group on all disease-related, (neuro)psychological and, work contextual factors. The people who quit participating showed worse scores on anxiety, depression, fatigue, workplace support, both objective and cognitive complaints and more frequently used emotion-oriented coping (data not shown). There were no differences in EDSS and the usage of other coping styles. In the current study, we tried to facilitate participation by using online questionnaires which participants could pause and proceed at their own time. Additionally, neurological and neuropsychological assessments were combined with their routine hospital visits, and people received regular updates on the study using a newsletter. However, the current amount of questionnaires was relatively extensive which may have required more individually tailored attention to increase the intrinsic motivation.

Fourthly, given that we did not include a control group we cannot affirm whether the current results are specific to the MS population or that similar trends may be observed in healthy people or people with other (chronic) illnesses.

Finally, only the SDMT was included as a measurement of objective cognitive functioning. However, objective cognitive functioning obviously entails more than information processing speed. Hence the current operationalization can be considered an oversimplification of the concept objective cognitive functioning. Additionally, time was positively significantly associated with the SDMT scores, reflecting learning effects.

## 5 Conclusion

At baseline, more symptoms of depression, a higher impact of fatigue, more cognitive complaints and less workplace support were related to a deterioration in employment status three years later. This suggests that timely identification of these factors is crucial to enable early intervention and prevent job loss in people with MS. MS-related disability, anxiety, objective cognition and coping styles were not related to a deterioration in employment status. Moreover, there were no differences in trajectories of disease-related, (neuro)psychological and work contextual factors between people with a stable and a deteriorated employment status. However, the current results are observed in a sample characterised by limited disability and stable clinical characteristics working in a Dutch setting, which needs to be taken into account when interpreting the results.

## CRediT authorship contribution statement

**Jeroen van Eijk**: Investigation, Resources, Writing – review & editing. **Stephan Frequin**: Investigation, Resources, Writing – review & editing. **Michiel Reneman**: Conceptualization, Funding acquisition, Writing – review & editing. **Martijn Beenakker**: Investigation, Resources, Writing – review & editing. **Oliver Gerlach**: Investigation, Resources, Writing – review & editing. **Karin van der Hiele**: Data curation, Funding acquisition, Project administration, Supervision, Validation, Writing – original draft, Writing – review & editing. **Jop Mostert**: Investigation, Resources, Writing – review & editing. **Mark de Rooij**: Conceptualization, Formal analysis, Methodology, Writing – review & editing. **Koen de Gans**: Investigation, Resources, Writing – review & editing. **Elske Hoitsma**: Investigation, Resources, Writing – review & editing. **Elianne van Egmond**: Conceptualization, Data curation, Formal analysis, Investigation, Methodology, Project administration, Validation, Writing – original draft, Writing – review & editing. **Huub Middelkoop**: Conceptualization, Funding acquisition, Supervision, Writing – review & editing. **Jac van der Klink**: Conceptualization, Funding acquisition, Writing – review & editing. **Wim Verhagen**: Investigation, Resources, Writing – review & editing. **Dennis van Gorp**: Data curation, Funding acquisition, Investigation, Project administration, Validation, Writing – review & editing. **Leo Visser**: Conceptualization, Funding acquisition, Investigation, Project administration, Resources, Supervision, Validation, Writing – review & editing. **Peter Jongen**: Writing – review & editing, Conceptualization, Funding acquisition.

## Funding

The MS@Work study was supported by 10.13039/501100001826ZonMw (TOP Grant, Project Number: 842003003), TeVa Pharmaceutical Industries, and Nationaal MS Fonds. Funding parties were not involved in any research activities.

## Declaration of Competing Interest

E.E.A. van Egmond, K. van der Hiele, M.J. de Rooij, D.A.M. van Gorp, J.J.L. van der Klink, M.F. Reneman, E.A.C. Beenakker, S.T.F.M. Frequin, K. de Gans, O.H.H. Gerlach, J.P. Mostert, and H.A.M. Middelkoop declare no conflict of interestP.J. Jongen received honoraria from Bayer Netherlands and Orikami Personalized Health Care for consultancy activities and is chairman of the MSmonitor Foundation.L.H. Visser received a research grant for the multicentre BIA study from Merck, received consultancy fees from Merck, Novartis and JanssenJ.J.J. van Eijk received consultancy fees and honoraria for lectures from Merck, Biogen, Novartis, Sanofi, Janssen and RocheE. Hoitsma received honoraria for lectures and advisory boards from Bayer, Biogen, Roche, Sanofi Genzyme, Merck Serono, Novartis and Teva.W.I.M. Verhagen received consultancy fees from Merck and Biogen

## Footnotes

1 Negative R values are set to zero.
