# Supplementary material for: Longitudinal determinants of employment status in people with relapsing-remitting multiple sclerosis
Source: IBRO Neurosci Rep. 2024 Apr 15;16:518–26. doi: 10.1016/j.ibneur.2024.04.002 (PMC11040135; doi:10.1016/j.ibneur.2024.04.002)
Supplement: Supplementary file 1 — Supplementary material [file mmc1.docx]

**Supplementary material Longitudinal determinants of employment status in people with relapsing-remitting Multiple Sclerosis**

**Results**

**Multilevel models for EDSS**

***UMM***

In the UMM for MS-related disability we included 632 observations. The intraclass correlation in the first model is 0.81.

***UGM***

Secondly, an UGM was fitted adding a fixed and random effect of time to the model. The UGM significantly improves the fit of the model with *χ^2^*(3)=46.82, *p*=3.79*10^-10^. The estimated value of the intercept is 2.07 and the estimated value of the slope of time is 0.05, indicating that the average value of EDSS at the start of the study is 2.07 and increases yearly by 0.05 (borderline significant, t(163.31)=1.97, p=0.0504). There is quite some variability at the start, that is, the variance of the intercept equals 1.22. The estimated variance of the slopes equals 0.05 and therefore some participants increase in EDSS while others decrease over the years. The estimated correlation between intercepts and slopes equals 0.18, indicating that those participants that have a higher value of EDSS at the start tend to increase faster.

***CGM (Model 3)***

Adding gender, age, education, disease duration, and their interaction with time to the model significantly improves the fit of the model (*χ^2^*(8)=31.56, *p*=0.000). These variables explain 15.6 percent of the variance of the intercepts, and 0 percent of the variance of the slopes^[[1]](#footnote-1)^. Age at baseline (t = 2.810, p = 0.006) and disease duration (t = 2.629, p = 0.009) were positively significantly related to MS-related disability at the start, no other main effects or interaction effects are statistically significant.

**Supplemental Table 1*.***

*Fixed effects for the CGM (Model 4) of MS-related disability (EDSS)*

|  | Estimate | Standard error | t-value | p-value |
| --- | --- | --- | --- | --- |
| Intercept | 0.44 | 0.57 | 0.78 | 0.437 |
| Time | 0.02 | 0.17 | 0.14 | 0.887 |
| DES | 0.31 | 0.20 | 1.59 | 0.113 |
| Age at baseline | 0.03 | 0.01 | 2.77 | **0.006** |
| Disease duration baseline | 0.04 | 0.02 | 2.52 | **0.013** |
| Education | 0.00 | 0.12 | 0.02 | 0.984 |
| Gender (male) | -0.09 | 0.22 | -0.41 | 0.680 |
| Time*DES | 0.10 | 0.06 | 1.78 | 0.076 |
| Time* Age | 0.00 | 0.00 | -0.38 | 0.708 |
| Time*Disease duration | 0.00 | 0.00 | 0.13 | 0.895 |
| Time*Education | 0.01 | 0.04 | 0.28 | 0.778 |
| Time*Gender (male) | 0.11 | 0.06 | 1.71 | 0.090 |

*Bold values indicate significant p-values.*

***Multilevel models for anxiety***

***UMM***

In the UMM for anxiety we included 650 observations. The intraclass correlation in the first model is 0.67.

***UGM***

Thereafter, an UGM was fitted adding a fixed and random effect of time to the model, significantly improving the fit of the model with *χ^2^*(3)=10.43, *p*=0.015. The estimated value of the intercept is 5.32 and the estimated value of the slope of time is -0.10, indicating that the average value of anxiety at the start of the study is 5.32 and decreases yearly by 0.10 although the time variable is not significant (t(165.24)=-1.41, *p*=0.16). There is quite some variability at the start, that is, the variance of the intercept equals 7.05. The variance of the slopes equals 0.25. The estimated correlation between intercepts and slopes equals -0.32, indicating that those participants that have a higher value of anxiety at the start tend to decrease faster.

***CGM (Model 3)***

Fitting a CGM (Model 3; including gender, age, education, disease duration, and their interaction with time) did not improve the model fit *χ^2^*(8)=11.61, *p*=0.169. The CGM explained no variation in intercepts, but did explain 5% variance of the slopes. The interaction between time and gender was positively significantly associated with anxiety (*p*=0.008).

*Supplemental Table 2.*

**Fixed effects for the CGM (Model 4) of anxiety**

|  | Estimate | Standard error | t-value | p-value |
| --- | --- | --- | --- | --- |
| Intercept | 7.08 | 1.56 | 4.54 | **1.080*10^-05^** |
| Time | 0.00 | 0.46 | -0.02 | 0.983 |
| DES | 0.00 | 0.54 | 0.01 | 0.990 |
| Age at baseline | -0.02 | 0.03 | -0.78 | 0.438 |
| Disease duration baseline | -0.02 | 0.04 | -0.57 | 0.572 |
| Education | -0.25 | 0.34 | -0.73 | 0.468 |
| Gender (male) | -0.17 | 0.60 | -0.28 | 0.781 |
| Time*DES | 0.20 | 0.16 | 1.26 | 0.210 |
| Time* Age | 0.00 | 0.01 | -0.49 | 0.624 |
| Time*Disease duration | 0.00 | 0.01 | -0.12 | 0.917 |
| Time*Education | -0.02 | 0.10 | -0.16 | 0.870 |
| Time*Gender (male) | 0.48 | 0.17 | 2.80 | **0.006** |

*Bold values indicate significant p-values.*

***Multilevel models for depression***

***UMM***

In the UMM for depression 650 observations were included. The intraclass correlation for depression is 0.66.

***UGM***

Secondly, an UGM was fitting adding a fixed and random effect of time to the equation, significantly improving the fit of the model with *χ^2^*(3)=25.87, *p*=1.02*10^-5^. The estimated value of the intercept is 5.16 and the estimated value of the slope of time is 0.11, indicating that the average value of depression at the start of the study is 5.16 and increases yearly by 0.11 (t(162.6) = 1.65, p = 0.10). The variance of the intercepts equals 5.16, while that of the slopes is 0.33. The estimated correlation between intercepts and slopes equals -0.21, indicating that those participants that have a lower value of depression at the start tend to increase faster.

***CGM (Model 3)***

Adding gender, age, education, disease duration, and their interaction with time to the model did not significantly improve the fit of the model (*χ^2^*(8)=11.96, *p*=0.153). These four variables did not explain any variance in the intercepts (0%), but a little of the slopes (5%)

***Multilevel models for the impact of fatigue***

***UMM***

In the UMM for fatigue we included 650 observations. The intraclass correlation for fatigue is 0.77.

***UGM***

Thereafter, an UGM was fitted adding a fixed and random effect of time to the model did not significantly improve the fit of the model with *χ^2^*(3)=5.57, *p*=0.135. The estimated value of the intercept is 33.73 and the estimated value of the slope of time is -0.24, t(161.641)=-0.84, p=0.0401). There is quite some variability at the start, that is, the variance of the intercept equals 183.31. The estimated variance of the slopes is 3.31. The estimated correlation between intercepts and slopes equals -0.12, indicating that those participants that have a higher value of fatigue at the start tend to decrease faster.

***CGM (Model 3)***

Adding gender, age, education, disease duration, and their interaction with time to the model did not significantly improve the fit of the model (*χ^2^*(8)=8.29, *p*=0.406). These four variables did explain some variance in the intercepts (2%), but not in the slopes (0%).

***Multilevel models for cognitive complaints***

***UMM***

In the UMM for cognitive complaints 650 observations were included. The intraclass correlation of cognitive complaints is 0.75.

***UGM***

Secondly, a UGM was fitted adding a fixed and random effect of time to the model. The UGM significantly improves the fit of the model with *χ^2^*(3)=11.43, *p*=0.010. The estimated value of the intercept is 22.74 and the estimated value of the slope of time is 0.23, indicating that the average value of cognitive complaints at the start of the study is 22.74 and increases yearly by 0.23. The increase was not significant t(153.07)=1.09, *p*=0.275). The variances of intercepts and slopes are 83.33 and 2.46, respectively. The estimated correlation between intercepts and slopes equals -0.10, indicating that those participants that have a higher value of cognitive complaints at the start tend to decrease faster.

***CGM (Model 3)***

Adding gender, age, education, disease duration, and their interaction with time to the model did not significantly improve the fit of the model (*χ^2^*(8)=12.29, *p*=0.139). These four variables did explain some variance in the intercepts (2%), but not in the slopes (0%). The interaction between time and age was significantly associated with cognitive complaints (*p*=0.038).

***Multilevel models for objective cognition***

***UMM***

In the UMM for supportive work place we included 656 observations. The intraclass correlation is 0.82.

***UGM***

Secondly, a UGM was fitted adding a fixed and random effect of time to the model. The UGM did significantly improve the fit of the model with *χ^2^*(3)=30.46, *p*=1.11*10^-06^. The estimated value of the intercept is 54.77 and the estimated value of the slope of time is 0.53. There is quite some variability at the start, that is, the variance of the intercept equals 62.27. The estimated variance of the slopes is 1.36. The estimated correlation between intercepts and slopes equals 0.07, indicating that those participants that have a higher value of workplace support at the start tend to increase faster.

***CGM (Model 3)***

Adding gender, age, education, disease duration, and their interaction with time to the model did significantly improve the fit of the model (*χ^2^*(8)=40.62, *p*=2.46*10^-06^). These four variables did explain some variance in the intercepts (15%), and in the slopes 6%). Age and the interaction between time and age were significantly associated with objective cognition (*p*=0.040 and *p*=0.025 respectively).

**Supplementary Table 3.**

*Fixed effects for the CGM (Model 4) of objective cognition*

|  | Estimate | Standard error | t-value | p-value |
| --- | --- | --- | --- | --- |
| Intercept | 59.91 | 4.06 | 14.75 | **<2*10^-06^** |
| Time | 2.07 | 1.02 | 2.03 | **0.044** |
| DES | -1.73 | 1.40 | -1.23 | 0.221 |
| Age at baseline | -0.16 | 0.08 | -2.04 | **0.043** |
| Disease duration baseline | -0.21 | 0.11 | -1.89 | 0.061 |
| Education | 1.74 | 0.88 | 1.97 | 0.0504 |
| Gender (male) | -2.96 | 1.57 | -1.88 | 0.062 |
| Time*DES | -0.41 | 0.35 | -1.18 | 0.240 |
| Time* Age | -0.04 | 0.02 | -2.24 | **0.026** |
| Time*Disease duration | 0.04 | 0.03 | 1.66 | 0.098 |
| Time*Education | 0.06 | 0.22 | 0.25 | 0.803 |
| Time*Gender (male) | -0.43 | 0.39 | 1.12 | 0.267 |

***Multilevel models for supportive work place***

***UMM***

In the UMM for supportive work place we included 649 observations. The intraclass correlation is 0.43.

***UGM***

Secondly, a UGM was fitted adding a fixed and random effect of time to the model. The UGM did not significantly improve the fit of the model with *χ^2^*(3)=3.95, *p*=0.267. The estimated value of the intercept is 10.44 and the estimated value of the slope of time is -0.45. There is quite some variability at the start, that is, the variance of the intercept equals 101.92. The estimated variance of the slopes is 5.82. The estimated correlation between intercepts and slopes equals -0.23, indicating that those participants that have a higher value of workplace support at the start tend to decrease faster.

***CGM (Model 3)***

Adding gender, age, education, disease duration, and their interaction with time to the model did not significantly improve the fit of the model (*χ^2^*(8)=7.94, *p*=0.440). Model 3 did not explain any variance in the intercepts (0%) or slopes (0%).

***Multilevel models for task-oriented coping***

***UMM***

In the UMM for task-oriented coping we included 650 observations. The intraclass correlation is 0.60.

***UGM***

Secondly, a UGM was fitted adding a fixed and random effect of time to the model. The UGM significantly improved the fit of the model with *χ^2^*(3)=9.59, *p*=0.022. The estimated value of the intercept is 59.25 and the estimated value of the slope of time is -0.25, indicating that the average value of task-oriented coping at the start of the study is 59.25 and decreases yearly by 0.25 (even though the slope was not significant, t(166.61)=-1.12, p=0.264). The variance of the intercepts and slopes are 49.03 and 2.45, respectively. The estimated correlation between intercepts and slopes equals -0.17, indicating that those participants that have a higher value of task-oriented coping at the start tend to decrease faster.

***CGM (Model 3)***

Adding gender, age, education, disease duration, and their interaction with time to the model significantly improves the fit of the model (*χ^2^*(8)=24.20, *p*=0.002). These variables explain 11 percent of the variance of the intercepts, and 0 percent of the variance of the slopes. Educational level (t = 3.43, p = 0.000) was significantly associated to task-oriented coping at the start, no other main effects or interaction effects are statistically significant.

**Supplemental Table 4.**

*Fixed effects for the CGM (Model 4) of task oriented coping*

|  | Estimate | Standard error | t-value | p-value |
| --- | --- | --- | --- | --- |
| Intercept | 47.36 | 4.14 | 11.45 | **2*10^-16^** |
| Time | 0.99 | 1.51 | 0.66 | 0.513 |
| DES | -0.12 | 1.43 | -0.07 | 0.941 |
| Age at baseline | 0.06 | 0.08 | 0.75 | 0.457 |
| Disease duration baseline | 0.21 | 0.11 | 1.88 | 0.06 |
| Education | 3.43 | 0.90 | 3.82 | **0.000** |
| Gender (male) | -0.92 | 1.58 | -0.58 | 0.562 |
| Time*DES | -0.73 | 0.51 | -1.43 | 0.156 |
| Time* Age | -0.00 | 0.03 | -0.16 | 0.874 |
| Time*Disease duration | -0.03 | 0.04 | -0.84 | 0.402 |
| Time*Education | -0.18 | 0.32 | -0.55 | 0.586 |
| Time*Gender (male) | -1.02 | 0.56 | -1.80 | 0.073 |

***Multilevel models for emotion-oriented coping***

***UMM***

In the UMM for emotion-oriented coping we included 650 observations. The intraclass correlation is 0.62.

***UGM***

Secondly, a UGM was fitted adding a fixed and random effect of time to the model. The UGM significantly improved the fit of the model with *χ^2^*(3)=31.39, *p*=7.03*10^-16^. The estimated value of the intercept is 35.53 and the estimated value of the slope of time is -1.13, indicating that the average value of task-oriented coping at the start of the study is 35.53 and significantly decrease yearly by 1.13 ( t(160.76)=-4.65, *p*=6.87*10^-6^). The variance of the intercepts and slopes are 66.58 and 2.54, respectively. The estimated correlation between intercepts and slopes equals -0.11, indicating that those participants that have a higher value of emotion-oriented coping at the start tend to decrease faster.

***CGM (Model 3)***

Adding gender, age, education, disease duration, and their interaction with time to the model did not significantly improve the fit of the model (*χ^2^*(8)=4.75, *p*=0.784). Model three did not explain variance in either the intercepts or the slopes compared to the UGM (both 0%).

**Supplemental Table 5.**

*Fixed effects for the CGM (Model 4) of emotion oriented coping*

|  | Estimate | Standard error | t-value | p-value |
| --- | --- | --- | --- | --- |
| Intercept | 32.66 | 4.98 | 6.55 | **6.92*10^-10^** |
| Time | 0.32 | 1.69 | 0.19 | 0.852 |
| DES | -0.84 | 1.72 | -0.49 | 0.624 |
| Age at baseline | 0.13 | 0.09 | 1.38 | 0.169 |
| Disease duration baseline | -0.17 | 0.13 | -1.27 | 0.206 |
| Education | -0.32 | 1.08 | -0.30 | 0.767 |
| Gender (male) | -2.04 | 1.90 | -1.07 | 0.287 |
| Time*DES | 0.39 | 0.57 | 0.68 | 0.497 |
| Time* Age | -0.03 | 0.03 | -0.87 | 0.388 |
| Time*Disease duration | 0.01 | 0.04 | 0.27 | 0.791 |
| Time*Education | -0.25 | 0.36 | -0.69 | 0.494 |
| Time*Gender (male) | 0.54 | 0.63 | 0.87 | 0.387 |

***Multilevel models for avoidance oriented coping***

***UMM***

In the UMM for avoidance oriented coping we included 650 observations. The intraclass correlation is 0.57.

***UGM***

Secondly, a UGM was fitted adding a fixed and random effect of time to the model. The UGM did not significantly improved the fit of the model with *χ^2^*(3)=6.66, *p*=0.084. The estimated value of the intercept is 46.09 and the estimated value of the slope of time is -0.39. The variance of the intercepts and slopes are 47.41 and 1.30, respectively. The estimated correlation between intercepts and slopes equals 0.10, indicating that those participants that have a higher value of avoidant coping at the start tend to increase faster.

***CGM (Model 3)***

Adding gender, age, education, disease duration, and their interaction with time to the model did significantly improve the fit of the model (*χ^2^*(8)=27.62, *p*<0.001). These four variables did explain 1% of the variance of the intercepts and 39% variance of the slopes. Gender, the interaction between time and diagnosis duration and the interaction between time and gender were significantly related to avoidance-oriented coping (*p*=0.046, *p*=0.050 and *p*=0.003 respectively).

**Supplemental Table 6.**

*Fixed effects for the CGM (Model 4) of avoidance oriented coping*

|  | Estimate | Standard error | t-value | p-value |
| --- | --- | --- | --- | --- |
| Intercept | 47.89 | 4.42 | 10.83 | **2*10^-16^** |
| Time | 0.13 | 1.52 | 0.08 | 0.934 |
| DES | 1.14 | 1.52 | 0.75 | 0.456 |
| Age at baseline | 0.02 | 0.08 | 0.27 | 0.786 |
| Disease duration baseline | -0.11 | 0.12 | -1.01 | 0.31 |
| Education | -0.65 | 0.96 | -0.68 | 0.497 |
| Gender (male) | -3.24 | 1.69 | -1.92 | 0.057 |
| Time*DES | -0.55 | 0.51 | -1.06 | 0.290 |
| Time* Age | -0.02 | 0.03 | -0.57 | 0.571 |
| Time*Disease duration | 0.08 | 0.04 | 2.04 | **0.043** |
| Time*Education | 0.02 | 0.32 | 0.05 | 0.959 |
| Time*Gender (male) | -1.78 | 0.57 | -3.15 | **0.002** |

1. Negative R^2^ values are set to zero [↑](#footnote-ref-1)
